# Insertion Sequences Determine Plasmid Adaptation to New Bacterial Hosts

**DOI:** 10.1128/mbio.03158-22

**Published:** 2023-04-25

**Authors:** Emilia Wedel, Cristina Bernabe-Balas, Manuel Ares-Arroyo, Natalia Montero, Alfonso Santos-Lopez, Didier Mazel, Bruno Gonzalez-Zorn

**Affiliations:** a Antimicrobial Resistance Unit (ARU), Facultad de Veterinaria and Centro de Vigilancia Sanitaria Veterinaria (VISAVET), Universidad Complutense de Madrid, Madrid, Spain; b Institut Pasteur, Université de Paris Cité, CNRS UMR3525, Unité de Plasticité du Génome Bactérien, Département Génomes et Génétique, Paris, France; The University of Edinburgh

**Keywords:** antimicrobial resistance, host range, insertion sequences, mutlicopy plasmids, plasmid-host adaptation

## Abstract

Plasmids facilitate the vertical and horizontal spread of antimicrobial resistance genes between bacteria. The host range and adaptation of plasmids to new hosts determine their impact on the spread of resistance. In this work, we explore the mechanisms driving plasmid adaptation to novel hosts in experimental evolution. Using the small multicopy plasmid pB1000, usually found in *Pasteurellaceae*, we studied its adaptation to a host from a different bacterial family, Escherichia coli. We observed two different mechanisms of adaptation. One mechanism is single nucleotide polymorphisms (SNPs) in the origin of replication (*oriV*) of the plasmid, which increase the copy number in E. coli cells, elevating the stability, and resistance profile. The second mechanism consists of two insertion sequences (ISs), IS*1* and IS*10*, which decrease the fitness cost of the plasmid by disrupting an uncharacterized gene on pB1000 that is harmful to E. coli. Both mechanisms increase the stability of pB1000 independently, but only their combination allows long-term maintenance. Crucially, we show that the mechanisms have a different impact on the host range of the plasmid. SNPs in *oriV* prevent the replication in the original host, resulting in a shift of the host range. In contrast, the introduction of ISs either shifts or expands the host range, depending on the IS. While IS*1* leads to expansion, IS*10* cannot be reintroduced into the original host. This study gives new insights into the relevance of ISs in plasmid-host adaptation to understand the success in spreading resistance.

## INTRODUCTION

The continuous emergence and increase of antimicrobial resistance over the last decades threaten our global health system and challenge the effective treatment of bacterial infections (1). Plasmids are key contributors to the dissemination of antibiotic resistance genes via horizontal gene transfer (2, 3). Most of them carry accessory genes that can provide benefits to the bacterial host in certain environments, among which are the antibiotic resistance genes (4). However, plasmids, together with other horizontally acquired determinants, are frequently associated with a high metabolic burden to the host cell (5–7). Several origins related to this fitness cost have been proposed, although the underlying molecular mechanisms are not completely understood. Among these fitness cost origins are the disruption of essential genes and cellular networks via the insertion of plasmid genes into the host genome, the replication and expression of plasmid genes, which can induce cytotoxic effects, and the sequestration of limited cell resources (5, 6).

Given this high cost, plasmids are expected to be either (i) lost in the absence of positive selection or (ii) partially integrated into the chromosome when genes encoding beneficial traits, such as antibiotic resistance enzymes, are selected. Therefore, it is challenging to understand how plasmids can survive and persist in bacterial populations in the absence of positive selection, which is known as “the plasmid paradox” (8–10). Nevertheless, recent works have shown an amelioration of the burden imposed by replicons driven by plasmid-host coevolutionary processes, leading to an increased long-term persistence of plasmids (8, 11–14). The genetic analysis of the coevolved bacteria and plasmids has revealed that the adaptation can be attributed to the chromosome (12, 15) or to the plasmid (13, 16, 17) independently, although in many cases it occurs due to a coadaptive effect of both bacteria and plasmid (11, 18, 19). When associated with an adaptive process of plasmids, the cost of the newly acquired plasmid has been described to be compensated for by mutations in the replication machinery (13), by the acquisition of a putative postsegregational killing system encoded in a transposon (18), by reducing the expression of the highly costly conjugative machinery (20), or by the deletion of a highly costly region of the plasmid (16). Moreover, ISs have been shown to play an important role in plasmid-host adaptation (21–23). For example, IS insertions in the chromosomal gene *ompF* of Escherichia coli cells, leading to reduced permeability and thus reduced antibiotic uptake, have been described to promote cost-reducing mutations affecting a plasmid-encoded tetracycline efflux pump (21, 22). Occasionally, the evolved plasmids impose a reduced burden not only to the ancestral host but also to alternative naive species (18), showing a generalized adaptive pattern which leads to an expansion of the host range of the plasmids.

With a few exceptions (15, 17), most of the studies and theoretical models of plasmid persistence focus on conjugative plasmids, which bear all the machinery necessary for their own transfer to new bacterial hosts and may differ from plasmid-bacterial adaptations of nonconjugative plasmids. These plasmids are usually smaller (less than 30 kb) and are present in a higher number of copies than conjugative plasmids. They also lack postsegregational killing systems, and it has been widely accepted that they stochastically divide into the two daughter cells during cell division, although recent studies show cluster distribution of some small plasmids in the cell poles (24, 25). Nonetheless, together with the plasmid fitness cost, the plasmid copy number is a determinant factor in the stability of nonconjugative multicopy plasmids.

Within the small nonconjugative plasmids, ColE1-like plasmids from *Pasteurellaceae* are strongly associated with antimicrobial resistance genes (26). It has been described that these plasmids are able to compensate for their biological cost through adaptive evolution, but their ability to adapt to other bacterial families has not yet been described (27). pB1000 is a small (4.6 kb) ColE1-type plasmid which has been described only in the *Pasteurellaceae* family. To date, it has been found in Haemophilus parasuis, Haemophilus influenzae, Pasteurella multocida, [Haemophilus] ducreyi (28–31) and Pasteurella aerogenes (GenBank accession number JQ773457). It encodes the beta-lactamase *bla*_ROB-1_ and confers high-level resistance to ampicillin and other beta-lactams (30). It has been well characterized in H. influenzae, where it shows an average copy number of 44.5 and a fitness disadvantage of 6 to 9% over ca. 10 generations (27, 30). pB1000 is stably maintained in H. influenzae and P. multocida for at least 50 to 200 generations in the absence of positive selection (27, 29). Moreover, in a previous study we were able to mobilize pB1000 into E. coli by transformation and conjugation using the conjugative machinery of a coresident plasmid (29). However, the plasmid was highly unstable and completely lost in the new bacterial host after 30 generations (29).

Insertion sequences (ISs) are also very common mobile elements in bacterial genomes (32). They are transposable elements that are composed of a transposase gene that catalyzes the transposition in a series of complex steps, including DNA cleavage and integration, and are flanked by inverted terminal repeats (33). They have been described to play a role in gene expression, fitness increase (34), and environmental adaptation (35). Apart from transposition within the genome, insertion sequences are able to incorporate genes from a vector into the chromosome, contributing to horizontal gene transfer, and have been described to be a vehicle of antimicrobial resistance spread (36). In addition to the ability to displace genes, ISs have also been shown to disrupt harmful genes, displaying a defensive function in bacteria (37).

In this work, we studied the adaptation of pB1000 to the novel host E. coli using experimental evolution. We observe that single nucleotide polymorphisms (SNPs) in the origin of replication of the plasmid lead to a higher copy number, whereas the acquisition of ISs decreases the fitness cost of the plasmid. While the mutations in the origin of replication impede the persistence of the plasmid in its parental host H. influenzae, the type of IS is critical in determining whether the plasmid undergoes a shift or an expansion of the host range. As both described mechanisms, SNPs and ISs, increase the stability of pB1000, but only the combination allows long-term maintenance, we show how the interplay of different evolutionary mechanisms results in successful plasmid adaptation, giving novel evidence of the role of compensatory evolution in the persistence of plasmids in bacterial populations.

## RESULTS AND DISCUSSION

### Plasmid-host coevolution drastically improves the stability of pB1000 in E. coli.

**FIG 1:**
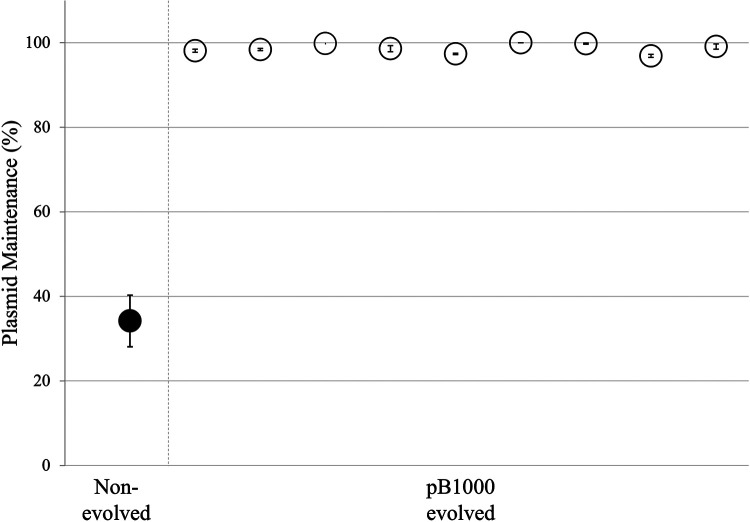
Plasmid maintenance (%) was measured for 10 days (~100 generations) or until the plasmid was completely lost from the population. The black sphere illustrates the ancestral plasmid in the ancestral strain, and the white spheres illustrate the evolved plasmids in the coevolved E. coli populations. For each plasmid-host combination, at least 3 independent replicates were analyzed. The standard errors are also indicated.

The plasmid pB1000 has been found in several species of the *Pasteurellaceae* family (28, 29), and it is stably maintained in P. multocida and H. influenzae (27, 29). However, it was highly unstable and rapidly lost when introduced into the novel host E. coli DH5α (Fig. 1), corresponding to previously published data (29). Further studies have shown increased plasmid persistence and stability through compensatory evolution (11–16, 18). To investigate the capacity of pB1000 to adapt to novel hosts, we conducted experimental evolution assays of pB1000 in E. coli. In two independent experiments we propagated a total of 9 lineages of E. coli DH5α bearing pB1000 over 500 generations with a daily 1:1,000 dilution transfer and forced the plasmid to be maintained by adding 50 mg/L of ampicillin to the medium.

At the end of the plasmid-host evolutions we tested for plasmid persistence. To do so, we analyzed the maintenance rate of pB1000 in the absence of ampicillin by calculating the plasmid loss rate per 10 generations (see Materials and Methods). In all nine populations the evolved plasmids were significantly more stable than in the ancestral E. coli (Fig. 1). For all of them, more than 90% of the colonies maintained the plasmid for at least 100 generations, suggesting that the pB1000-E. coli coevolution improved plasmid stability in a novel host.

### The adaptive evolution of pB1000 in E. coli is mediated by the acquisition of insertion sequences and SNPs.

**FIG 2:**
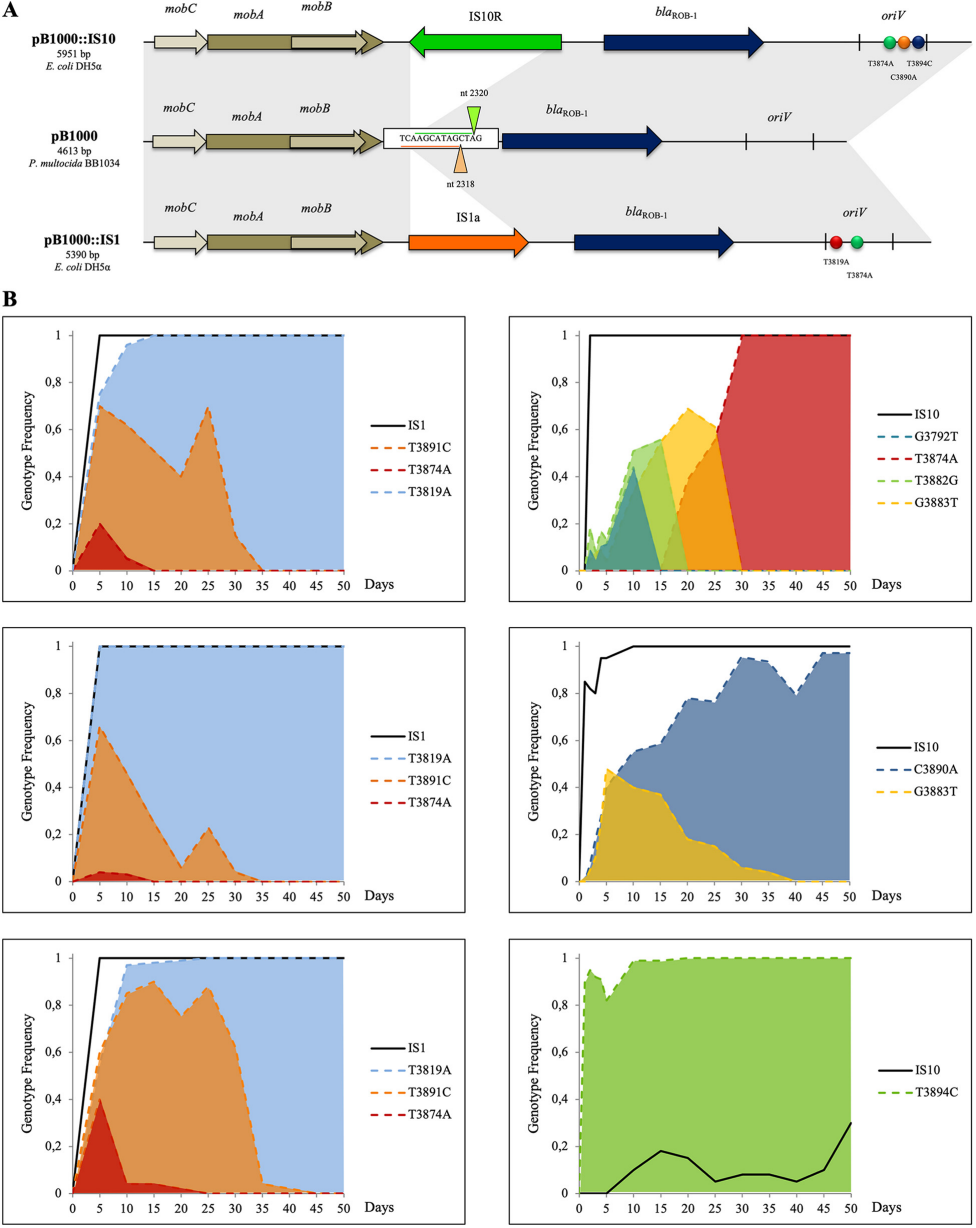
(A) Genetic structures of pB1000 and the evolved plasmids. The gray shadows indicate sequence identity. The arrows mark ORFs, with their heads indicating the direction of transcription. The *oriV* of the plasmids are delimited by vertical bars. The triangles indicate the positions where the ISs transposed, whereas circles indicate SNPs that were present at the end of the evolutions (not necessarily in the same plasmid). (B) Genetic dynamics of pB1000 in six evolved replicate populations. The left and right panels correspond to three replicate lineages, originating from a single colony. The colored areas show the frequencies of different genotypes over time. The black solid line represents the acquisition and frequency of IS*1* or IS*10* over time.

Several works have shown how compensatory mutations ameliorate the fitness cost of carrying a plasmid (9–13, 16). We therefore analyzed the sequences of the evolved plasmids to further investigate if any adaptive mutations could explain the observed improved persistence of pB1000 in E. coli. Before starting the evolution assays and during the experiments, plasmid DNA was extracted and analyzed (Fig. 2B) (see Materials and Methods).

In six of the nine lineages pB1000 acquired the insertion sequence 1 (IS*1a*) (GenBank accession number: X52534) from the E. coli chromosome (Table 1). This insertion sequence is 768 bp long and expresses the InsAB′ transposase, necessary and sufficient for its own transposition (38). When transposed, it generated a 9-bp direct repeat (CAAGCATAG) at both sides of the IS. Remarkably, in all the plasmids recovered from these six evolving replicates, IS*1* was inserted into the exact same position of pB1000, at nucleotide 2318 of its sequence (GenBank accession number: GU080062), located between the relaxase genes and the resistance gene *bla*_ROB-1_ (Fig. 2A).

**TABLE 1:** Plasmid genotypes obtained at day 50 of the 9 evolved lineages

Lineage	Plasmid size (bp)	Acquired IS	SNP in the putative *oriV*
1	5,390	IS*1a*	T3819A
2	5,390	IS*1a*	T3819A
3	5,390	IS*1a*	T3874A
4	5,390	IS*1a*	T3819A
5	5,390	IS*1a*	T3819A
6	5,390	IS*1a*	T3819A
7	5,951	IS*10R*	T3874A
8	5,951	IS*10R*	C3890A
9	5,951	IS*10R*	T3894C

In the other three populations, a different insertion sequence, the IS*10R* (GenBank accession number: AH003348), was mobilized from the chromosome into the plasmids. This 1,329-bp long IS, which also induced a 9-bp direct repeat (AGCTATGCT), was integrated at nucleotide 2320 of pB1000, just two nucleotides downstream of the insertion site of IS*1* in the previous assays (Fig. 2A).

Insertion sequences are known to circulate between plasmids and chromosomes (36). Both ISs that jumped to pB1000 are typically found in the E. coli genome, with a mean of 6 to 8 copies for IS*1* (39) and 2 to 4 copies for IS*10* (40). These ISs have recently been described to hold a defensive function against plasmids that encode a protein which is toxic for bacterial cells, by the transposition from E. coli host chromosomes to the damaging gene on the plasmid (37). Their transposition rates (per element per generation) differ from 2.67 × 10^−5^ for IS*1* (41) to 1 × 10^−4^ for IS*10* (42). While IS*1* does not have a defined target site, IS*10* preferentially inserts at a 9-bp symmetrical consensus sequence (NGCTNAGCN) (43), which matches the insertion site in pB1000 except for 1 nucleotide (AGCTA**T**GCT). The transposition of both insertion sequences into the same exact region of the plasmid throughout all the parallel evolutions suggests that the acquisition at this position plays a relevant role in the adaptive evolution and the increased persistence of pB1000 in E. coli.

In addition to the acquisition of ISs, we found single nucleotide polymorphisms (SNPs) between nucleotides 3792 and 3891 in the putative *oriV* of the evolved plasmids at the end of the assays (44) (Fig. 2A). Various SNPs and double alleles appeared at different times of the experimental evolutions, usually after the acquisition of the IS from the host chromosome (Fig. 2B). Nevertheless, after ~300 to 400 generations, during which the arising genotypes coexist in the population, only one mutation per lineage was fixed and established in the population (Table 1), presumably due to the highest benefit in terms of fitness/plasmid stability (45). The evolutionary process of lineages displayed in the left panels of Fig. 2B does not exclude the possibility that the original colony from which the three lineages were propagated already carried plasmids with the mutations and the insertion sequence. However, the fact that the mutations and IS insertion went from undetectable to rapidly increasing in frequency, ultimately being fixed in the population, highlights their role in increasing the fitness of the strains bearing the mutated pB1000 (46, 47).

Parallel evolution is one of the major signatures of adaptive evolution. When multiple independent populations acquire modifications in the same target, this provides strong evidence of selection of this trait (13, 47, 48). In our experimental evolution, we detected parallel evolution for the acquisition of mutations in the origin of replication and IS insertions upstream of the *bla*_ROB-1_ gene, suggesting that these mutagenic events are involved in the adaptive evolution of pB1000 to E. coli.

### The insertion sequences and the SNPs in the *oriV* mediate the stabilization of pB1000 in the new host.

**FIG 3:**
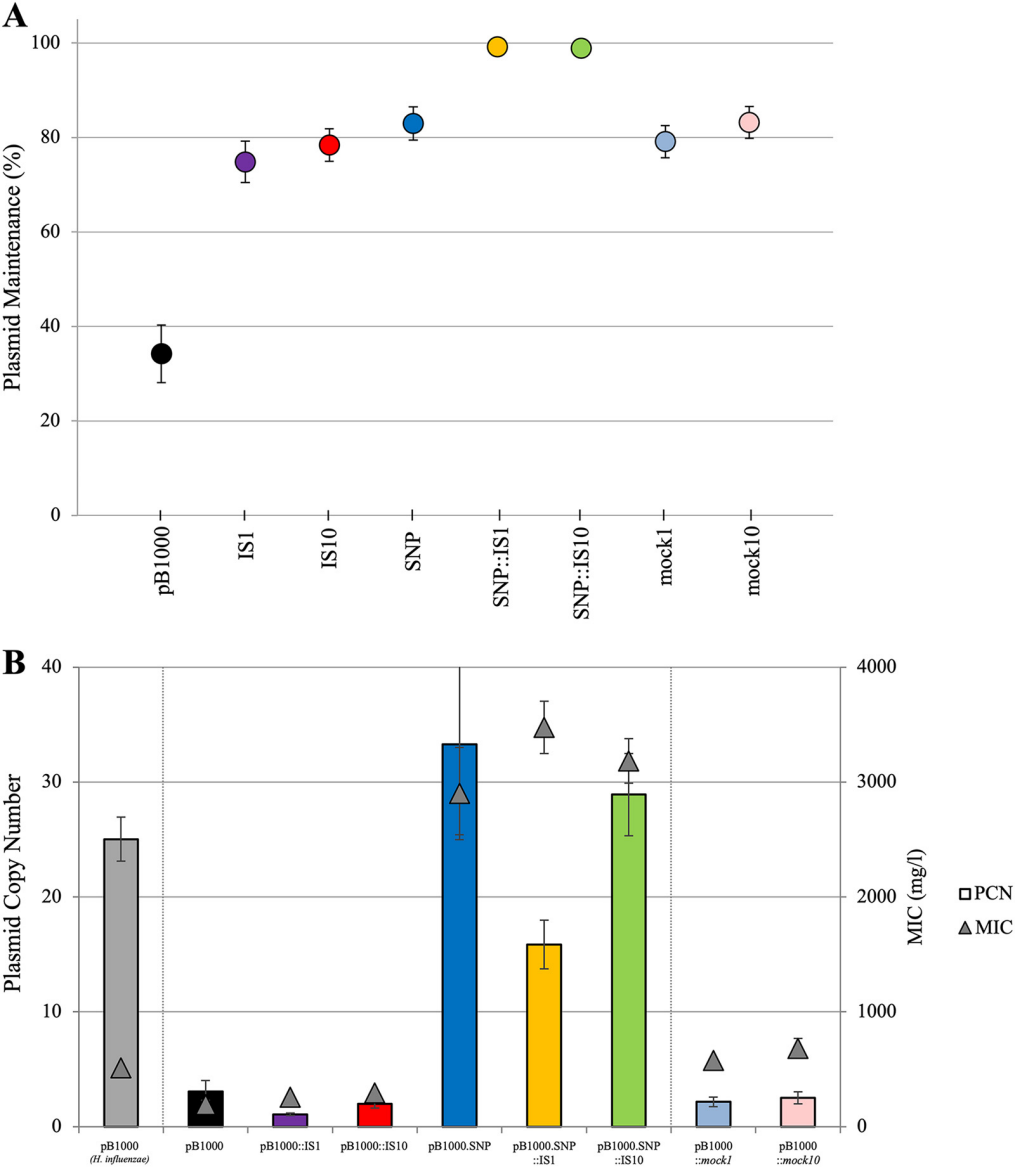
(A) Plasmid maintenance (%) of the seven plasmid variants in ancestral E. coli was measured for 10 days (~100 generations) or until the plasmid was completely lost from the population. The maintenance of the ancestral plasmid in E. coli is also shown. Data points represent the average of 4 to 9 replicates at each time point. (B) PCN (bars) and MIC (triangles) of the ancestral pB1000 and the plasmid variants in E. coli.

The acquisition of SNPs and ISs under selective pressure increases the stability of the evolved plasmids. To study the contribution of each adaptive step, we selected 5 plasmids obtained from the evolved lineages: two plasmids bearing each IS separately (pB1000::IS*1* and pB1000::IS*10*), one plasmid with SNP T3874A in the *oriV* (pB1000.SNP), and the combination of each IS and the SNP in the same plasmid (pB1000.SNP::IS*1* and pB1000.SNP::IS*10*). We chose SNP T3874A in the *oriV* because it arose in two different lineages and it was the only one present in combination with both ISs (Fig. 2). The obtained strains and the genotypes of the plasmid mutants are listed in Table 2. We then transformed these plasmids into the ancestral nonevolved E. coli and analyzed their maintenance rate without selective pressure (Fig. 3A).

**TABLE 2:** Bacterial strains used in this study

Strain	Plasmid	Resistance genes	Reference or accession no.
P. multocida BB1034	pB1000	*bla*_ROB-1_ (pB1000) *tet*(B) (pB1001)	29
	pB1001		
H. influenzae RdKW20	None	None	NC_000907
E. coli DH5α	None	None	NZ_JRYM00000000
Ec-pB1000	pB1000	*bla*_ROB-1_	This work
Ec-IS1	pB1000::IS*1*	*bla*_ROB-1_	This work
Ec-IS10	pB1000::IS*10*	*bla*_ROB-1_	This work
Ec-SNP	pB1000.SNP	*bla*_ROB-1_	This work
Ec-SNP/IS1	pB1000.SNP::IS*1*	*bla*_ROB-1_	This work
Ec-SNP/IS10	pB1000.SNP::IS*10*	*bla*_ROB-1_	This work
Ec-mock1	pB1000::mock1	*bla*_ROB-1_	This work
Ec-mock10	pB1000::mock10	*bla*_ROB-1_	This work
Ec-mock1’	pB1000::mock1′	*bla*_ROB-1_	This work
Ec-mock10’	pB1000::mock10′	*bla*_ROB-1_	This work

Plasmids bearing only an IS were significantly more persistent in the ancestral E. coli than pB1000, as the plasmid maintenance rate increased from 35.5% ± 5.1 standard error (SE) to 76.0% ± 3.3 (SE) for pB1000::IS*1* (two-sample *t* test: *P < *0.01) and to 80.0% ± 2.7 SE for pB1000::IS*10* (two-sample *t* test: *P < *0.01). The plasmid with the SNP in the *oriV* showed an even higher maintenance rate of 81.1% ± 2.3 SE relative to the ancestral pB1000 (two-sample *t* test: *P < *0.01). However, the highest maintenance rate was achieved by the two plasmid variants harboring both an IS and the SNP (99.5% ± 0.5 SE for pB1000.SNP::IS*1* and 99.3% ± 0.7 SE for pB1000.SNP::IS*10*). These values correspond to the same maintenance rates displayed by the nine evolved strains (Tukey’s honestly significant difference [HSD] *post hoc* test: *P = *0.07).

Our findings suggest that the plasmid-host adaptation of E. coli and pB1000 is mediated by the modifications in the plasmid. These modifications act independently in the adaptation of pB1000 to E. coli, but the combination of both variations is responsible for the increased plasmid maintenance of almost 100% of the replicon (Fig. 1).

### The combined effect of increasing the plasmid copy number and decreasing the fitness cost is responsible for the successful adaptation of pB1000 to E. coli.

ColE1-like plasmids are considered to be randomly partitioned during cell division, as no active partitioning systems are known to exist in them (49). Although it has recently been suggested that *oriV* may stabilize plasmids beyond adjusting their copy number (50), it is generally accepted that the lack of a segregation system makes the plasmid copy number (PCN) and the imposed fitness cost important factors involved in the plasmids’ cellular stability. Hence, to test if modifications of the PCN were involved in the increased stability of pB1000 in E. coli, we analyzed the copy number of each of the selected plasmid variants in nonevolved E. coli via quantitative PCR (see Materials and Methods).

While in our experiments the average PCN of pB1000 in its natural host, H. influenzae, is 25.0 ± 1.9 SE copies per bacteria, the PCN of pB1000 in E. coli is 3.1 ± 0.9 SE (Fig. 3B). This suggests a significant reduction of the PCN in the new host (two-sample *t* test: *P < *0.01). Likewise, plasmids bearing an IS (but no SNP in *oriV*) show the same PCN as the ancestral pB1000 in E. coli (1.1 ± 0.1 SE for IS*1* and 2.0 ± 0.4 SE for IS*10*) (IS*1*: two-sample *t* test: *P = *0.07; IS*10*: two-sample *t* test: *P = *0.32). However, all plasmids with the SNP in *oriV*, with or without an IS, showed an increase in the PCN compared to the plasmids without an SNP (two-sample *t* test: *P* = <0.01), with no significant differences among the three of them (33.3 ± 7.9 SE, 15.9 ± 2.1 SE, and 28.9 ± 3.6 SE copies for pB1000.SNP, pB1000.SNP::IS*1*, and pB1000.SNP::IS*10*, respectively) (one-way analysis of variance [ANOVA]: *F* = 3.1, *P = *0.07). Thus, we observe that the low PCN of pB1000 in E. coli presumably is a cause of its low stability, and the evolved and adapted pB1000 variants show an increased PCN through the acquisition of SNPs in *oriV*.

It has been shown before that beta-lactam resistance correlates linearly with the amount of beta-lactamase that is expressed (27, 51–53). Therefore, the increased PCN of the new pB1000 variants might generate an increased resistance phenotype in the new host. To test this hypothesis, we analyzed the resistance profile to ampicillin of the strains containing the selected plasmids (Fig. 3B). The MIC of E. coli with pB1000 was 192 mg/L, and it did not significantly increase in the presence of IS*1* (MIC = 256 mg/L, two-sample *t* test: *P = *0.16). IS*10* (294 mg/L, two-sample *t* test: *P = *0.02) shows a significantly higher MIC, and the three strains bearing the plasmids with a SNP (and consequently higher PCN) exhibited an over 10-fold significant increase in their ampicillin resistance phenotype compared to those lacking the SNP (two-sample *t* test: *P < *0.01). They reached MICs of 2,902 mg/L, 3,477 mg/L, and 3,185 mg/L for Ec-SNP, Ec-SNP/IS*1*, and Ec-SNP/IS*10*, respectively, with no significant difference among them (one-way ANOVA: *P = *0.36).

Mutations in different components of the replication machinery from ColE1-like plasmids have been described to affect the average plasmid copy number in the cell (54, 55). In our case, we show that the SNP T3874A, located within the putative *oriV* of pB1000, increases the PCN in E. coli by 25-fold (and subsequently the MIC), leading to an increase in the stability of the plasmid. In contrast, plasmids that bear only an IS had neither a modified copy number nor an increased resistance phenotype. Still, the acquisition of ISs led to higher plasmid stability than that of the ancestral pB1000, suggesting that an additional mechanism is responsible for their improved persistence.

Apart from their copy number, the stability of multicopy plasmids is highly influenced by the fitness burden they impose on the cell. Therefore, we analyzed the fitness cost of the ancestral pB1000 and the plasmids pB1000::IS*1* and pB1000::IS*10*, as all three of them exhibit the same PCN (~1.5 copies) but different maintenance rates (~40% pB1000 versus ~80% pB1000 with IS). The fitness cost was assessed by competition experiments between isogenic plasmid-bearing and plasmid-free E. coli strains (see Materials and Methods). The fitness of the plasmid-bearing E. coli was calculated using the selection coefficient (*s*) relative to the parental E. coli. While pB1000 produces a fitness cost of 5.7% ± 0.2 SE (*s* = −0.057) in its natural host, H. influenzae (27, 30), it has a much higher fitness impact on E. coli, with a cost of 77.3% ± 3.1 SE (*s* = −0.773). This would explain the rapid loss of the plasmid in the population. However, we observed an amelioration in the fitness cost imposed by pB1000 when bearing either IS*1* or IS*10* (*s* = −0.232 ± 0.047 SE for pB1000::IS*1*; *s* = −0.220 ± 0.039 SE for pB1000::IS*10*). These results indicate that the presence of either of the two ISs in pB1000 induced a significant 3-fold reduction of the biological burden (two-sample *t* test: *P* = <0.01 for pB1000::IS*1*; two-sample *t* test: *P* = <0.01 for pB1000::IS*10*).

Here, we have shown how, by two different genetic mechanisms, increasing the PCN via SNPs in *oriV* and reducing the plasmid burden through the acquisition of host-encoded ISs, pB1000 is able to adapt to a new host in which it was initially highly unstable. Both mechanisms partially increase the persistence of the plasmid, but only the combination of both allows the long-term maintenance of pB1000.

### The expression of a 42-amino acid peptide is responsible for the high instability of pB1000.

One question that arises from the previous results is, by which mechanism can the insertion sequences reduce the fitness cost of pB1000? Insertion sequences have been previously described to reduce the biological burden imposed by plasmids through the deletion of a highly costly region of the plasmid by transposition of the mobilizable element (16) or by mutating a harmful gene (37).

As mentioned before, both IS*1* and IS*10* transposed into the same region of the evolved pB1000, at nucleotides 2318 (IS*1*) and 2320 (IS*10*). Therefore, the observed amelioration of the fitness cost could also be due to changes in the expression of the *bla*_ROB-1_ gene by promoters encoded in the insertion sequences, as widely described in the bibliography (56, 57). However, the relatively long distance to the beginning of the coding sequence, the unchanged MIC levels of the strains with IS-bearing plasmids, and the acquisition of the ISs into the exact same position indicate that this region plays an important role in the fitness cost of the plasmids.

**FIG 4:**
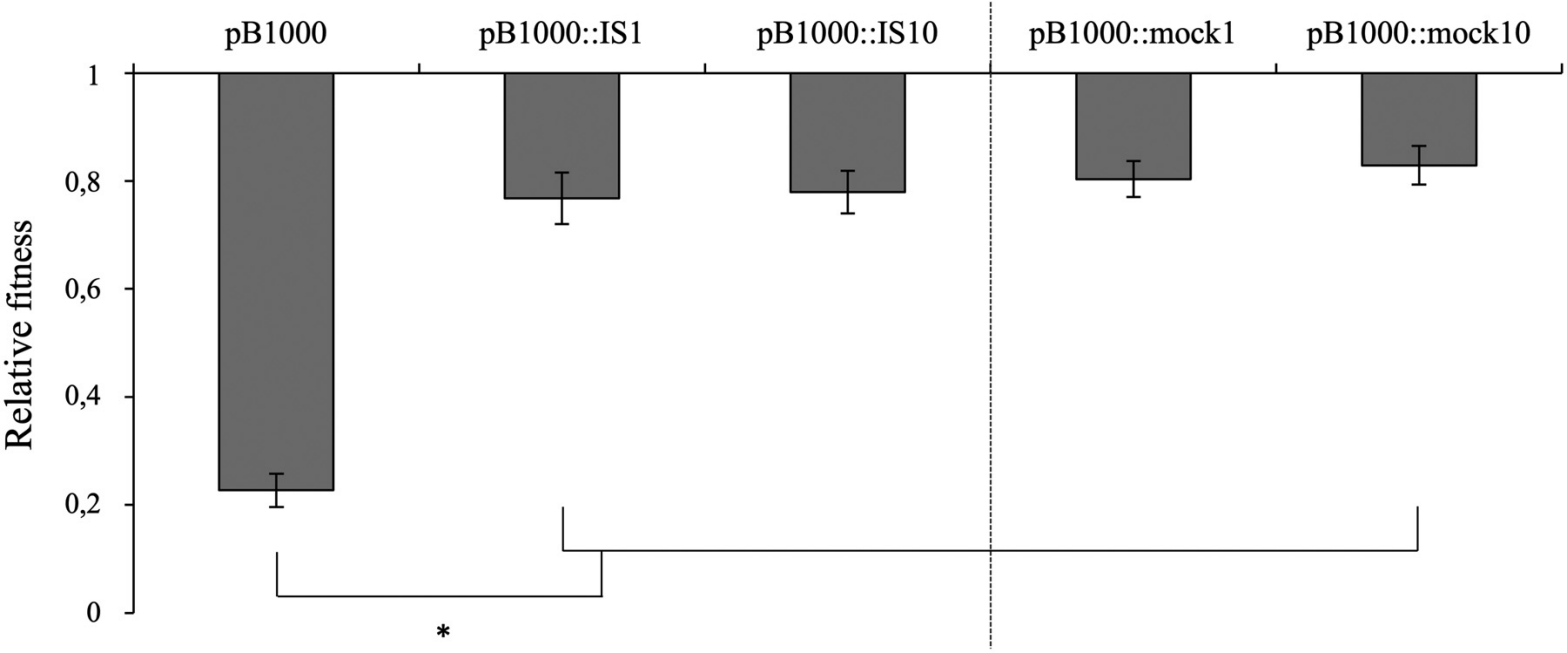
Increase in the relative fitness of the strains bearing pB1000 due to the acquisition of an insertion sequence or a mock fragment, compared to the E. coli strain carrying the original pB1000. The average and the standard errors of 6 independent measurements are represented. The bars and the asterisk indicate significant differences.

To investigate this hypothesis, we cloned two noncoding fragments with the same sizes as IS*1* and IS*10* into pB1000, into the same positions where each IS had transposed, resulting in the plasmids pB1000::mock1 and pB1000::mock10. We analyzed the maintenance rate and fitness cost of these two constructions. Remarkably, the results were similar to those obtained with the plasmids harboring the ISs. The maintenance rate was 79.1% ± 3.4 SE for pB1000::mock1 and 83.2% ± 3.4 SE for pB1000::mock10. The plasmids with mock fragments show the same stability as those with ISs (one-way ANOVA: *P = *0.52) (Fig. 3A). Regarding the fitness burden imposed by the plasmids, a decrease to the level of the cost conferred by pB1000::IS*1* and pB1000::IS*10* was observed (one-way ANOVA: *P = *0.76) (Fig. 4).

Together, these results suggest that the increased persistence of pB1000 bearing either an IS or a mock fragment is due to the disruption of an unknown genetic element located within this DNA region. An *in silico* analysis of the disrupted region in pB1000 revealed the presence of two antiparallel small open reading frames (ORFs), ORF3 (nucleotides [nt] 2296 to 2397 of pB1000) and ORF40 (nt 2402 to 2274 of pB1000), that were disrupted by the insertion of the mobile elements. To check if this region is specific to *Pasteurellaceae* and already described, we extracted the ORF40 and ORF3 regions and looked for them in the NCBI database conducting blast searches. Interestingly, the nucleotide sequences are found only in *Pasteurellaceae*, mainly in small plasmids that present very similar structures (see Fig. S1 in the supplemental material): pB1000 (GenBank accession number: GU080062), pB1000′ (GU080066), p1144 (MK203847), pJMA-1 (KP164834), pCCK343 (FR687372), pB1002 (EU283341), pAPP7_A (CP001094), pHS-Tet (AY862435), pMS2 (LC556098), p1712 (OL697394). Searching for possible encoded proteins, we only found a hypothetical protein described in a comparative genomic study of Actinobacillus pleuropneumoniae, also of the *Pasteurellaceae* family (58).

10.1128/mbio.03158-22.1 FIG S1 Comparison of the genomic composition of plasmids that carry the ORF40/ORF3 sequence (here labeled as “shyP”). The scale indicates the percentage of sequence identity. Gray illustrates sections that have the same orientation, and red represents inversions. Easyfig version 2.2.2 was used to create the comparison figure. Download FIG S1, TIF file, 3.0 MB. Copyright © 2023 Wedel et al. 2023 Wedel et al. https://creativecommons.org/licenses/by/4.0/ This content is distributed under the terms of the Creative Commons Attribution 4.0 International license.

To investigate the possible expression of small peptides by any of these two ORFs, we cloned them separately into the pBAD43 vector, whose arabinose P_BAD_ promoter allows tight control of expression (59). Then we analyzed the effects of expressing these ORFs on plasmid stability in the laboratory strain E. coli MG1655 (Fig. S2). E. coli populations were transformed with plasmids pBAD43, pBAD43::ORF40, and pBAD43::ORF3. Afterward, stability assays were performed, either expressing or repressing the different ORFs (Fig. S2). All plasmids were maintained in 100% of the E. coli populations for at least 80 generations, except pBAD43::ORF40 under inducing conditions. Lineages expressing ORF40 rapidly lost the plasmid after 10 generations. This result suggests that ORF40 is translated into a peptide that is toxic for E. coli and that its disruption by ISs allows pB1000 to be maintained longer in this novel host. To verify the expression of an ORF40 peptide, we first extracted proteins of E. coli at an optical density at 600 nm of 0.6 of populations in which ORF40 was expressed or not expressed and then performed Western blotting using the 3×Flag system (Sigma-Aldrich, USA) for protein detection (Fig. S3). The results confirmed that ORF40 encodes a small peptide of 42 amino acids. Because of the high number of hydrophobic amino acids in the peptide and because it was exclusively found in *Pasteurellaceae*, we named it peptide ShyP (small hydrophobic *Pasteurellaceae*) with its corresponding gene *shyP*. Further studies are needed to determine the role of ShyP in the *Pasteurellaceae* family.

10.1128/mbio.03158-22.2 FIG S2 Genetic constructions of the vector pBAD43 bearing ORF3 and ORF40 and stability of the constructions under repressing and inducing conditions. Download FIG S2, TIF file, 2.2 MB. Copyright © 2023 Wedel et al. 2023 Wedel et al. https://creativecommons.org/licenses/by/4.0/ This content is distributed under the terms of the Creative Commons Attribution 4.0 International license.

10.1128/mbio.03158-22.3 FIG S3 Western blot of protein extractions from E. coli cells. The columns show the positive (1) and negative (2) control for the Flag tag and extractions from cells grown either inducing (3) or repressing (4) the expression of ORF40FT at an OD_600_ of 0.6. Download FIG S3, TIF file, 2.9 MB. Copyright © 2023 Wedel et al. 2023 Wedel et al. https://creativecommons.org/licenses/by/4.0/ This content is distributed under the terms of the Creative Commons Attribution 4.0 International license.

### Expansion or shift in the host range of pB1000 after its adaptation.

Plasmid host range has been shown to evolve throughout adaptive evolution, leading to shifts (13) or even expansion (18) of the initial host range of the ancestral plasmid. In this work, we have shown how pB1000 is able to persist and achieve ~100% stability in E. coli through the acquisition of ISs and/or mutations in the origin of replication over an experimental evolution of 500 generations. To determine if these adaptive mutations changed the stability of pB1000 in its native host, we electroporated the plasmid variants bearing an IS, a mutation in *oriV* and a mock fragment (all the plasmid variants are listed in Table 3) into electrocompetent H. influenzae.

**TABLE 3:** Plasmid variants used in this study

Plasmid	Size (bp)	Insertion	SNP or bp addition	Reference or accession no.
pB1000	4,613	None	None	GU080062
pB1000.SNP	4,613	None	Mutation of nucleotide 3874 from thymine to adenine	This work
pB1000::IS*1*	5,390	IS*1*	None	This work
pB1000.SNP::IS*1*	5,390	IS*1*	Mutation of nucleotide 3874 from thymine to adenine	This work
pB1000::mock1	5,390	Fragment of the same size of IS*1*	None	This work
pB1000::mock1’	5,390	Without HindII target	Mutation of target GTCGAC to GTAGAC	This work
pB1000::IS*10*	5,951	IS*10*	None	This work
pB1000.SNP::IS*10*	5,951	IS*10*	Mutation of nucleotide 3874 from thymine to adenine	This work
pB1000::mock10	5,951	Fragment of the same size of IS*10*	None	This work
pB1000::mock10′	5,951	Without HindII target	Mutation of target GTCGAC to GTAGAC	This work
pBAD43	6,179	None	None	59
pBAD43::ORF3	6,281	ORF3	None	This work
pBAD43::ORF40	6,308	ORF40	None	This work
pBAD43::ORF40-3FT	6,374	ORF40-3FT	None	This work

Plasmids with the SNP T3874A, either alone or in combination with IS*1* or IS*10*, could not be transformed to H. influenzae. This suggests that the mutation in *oriV* prevents the successful replication of pB1000.SNP in the original host. Likewise, pB1000 plasmids bearing IS*10*, the mock1 fragment, or the mock10 fragment, could not be successfully transformed to H. influenzae. While this could indicate that the disruption of *shyP* additionally affects the host range of pB1000, we observed that pB1000::IS*1* was successfully electroporated into H. influenzae and was 100% maintained for at least 100 generations. An alternative hypothesis is that the ISs (and mocks) themselves are involved in the change of the host range of pB1000. The analysis of the nucleotide sequences of the IS*10* and the mock fragments revealed the target site of the restriction endonuclease HindII (5′-GTTGAC) (43). Since no HindII targets were found either in the original pB1000 or the IS*1* sequence, we hypothesize that the reason why no transformants with IS*10* or the mock fragments occur is because of the digestion of the plasmids upon electroporation into H. influenzae. To verify this, we mutated the HindII target sites of the mock sequences (5′-GTCGAC to 5′-GTAGAC), which resulted in successful transformation of pB1000::mock1′ and pB1000:mock10′ to H. influenzae, but not of pB1000::IS*10*. The pB1000::mock plasmids were maintained for at least 100 generations, with all of the colonies harboring a plasmid at the end of stability assays. We conclude that the sequence of the IS disrupting the *shyP* gene influences whether the plasmid adaptation leads to an expansion or to a shift in the host range and that targets of restriction endonucleases can play an important role in plasmid adaptation.

**FIG 5:**
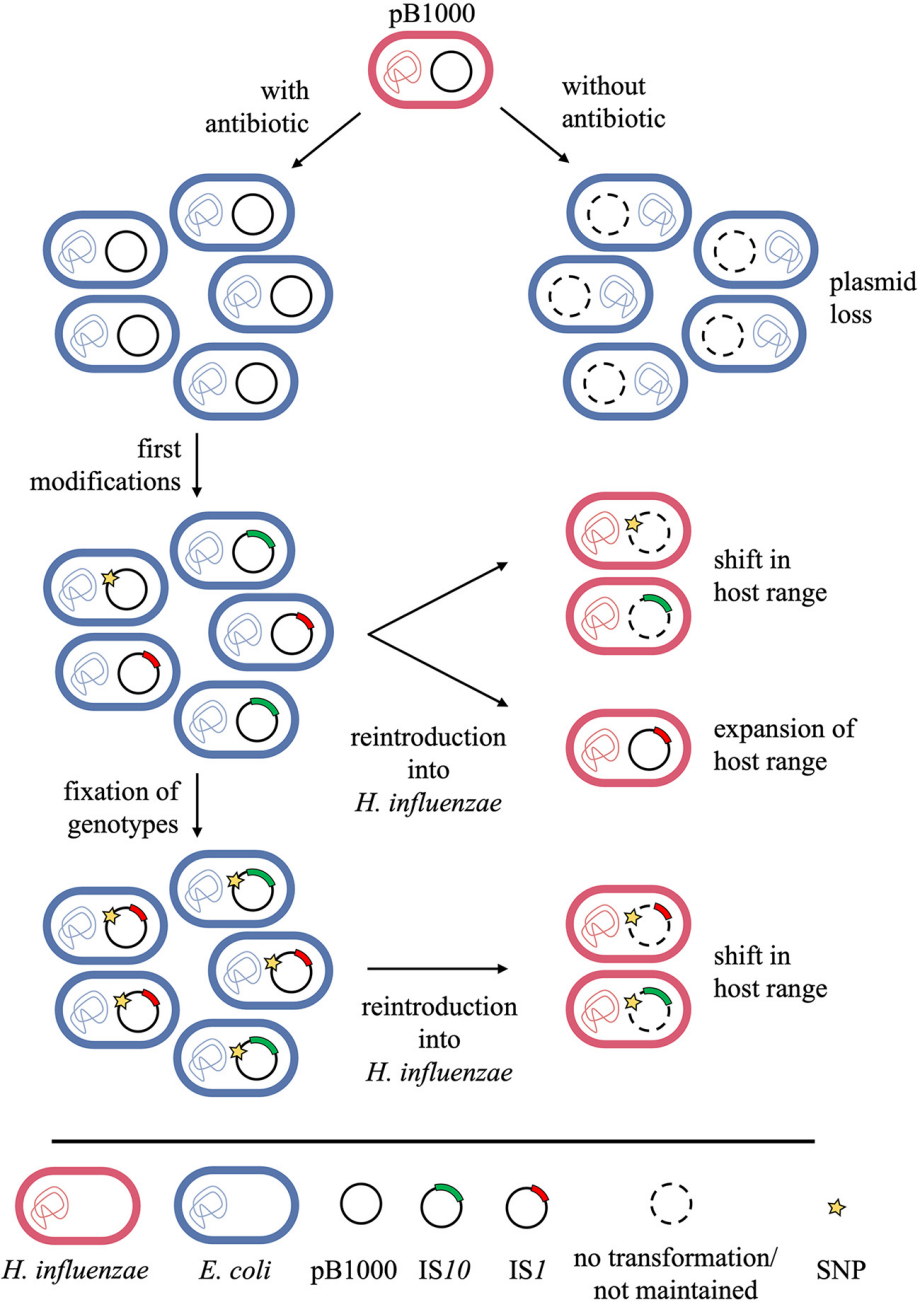
Model of the evolution assays of E. coli containing pB1000. Two different mechanisms of adaptation lead to either a shift of the host-range of the plasmid (SNPs, IS*10*) or to an expansion (IS*1*). Only the combination of IS and SNP lead to a plasmid stability of 100%.

Taken together, these results show two different evolutionary pathways of pB1000 adaptation to a novel species (Fig. 5). The first one, the acquisition of SNP T3874A in the *oriV* gene of the plasmid, allows pB1000 to persist longer in a new bacterial family but leads to the loss of its capacity to persist in its natural host. In the second pathway, on the other hand, pB1000 acquires an IS that disrupts a toxic peptide encoded in its sequence, increasing stability by decreasing the biological cost to the cell. Furthermore, depending on the IS itself, the disruption can lead to an expanded host range by increasing the maintenance in a new host while still being able to persist in the original host.

## MATERIALS AND METHODS

### Bacterial strains, culture conditions, and antibiotic susceptibility determination.

The bacterial strains and the plasmids used in this study are listed in Tables 2 and 3, respectively. P. multocida BB1034, from where pB1000 was obtained, was cultured on Columbia agar plus 5% sheep blood plates (bioMérieux, France). H. influenzae RdKW20, which was used for measuring the transformation rate, was cultured on chocolate agar PolyViteX plates (bioMérieux) and in Haemophilus test medium (HTM) broth (Francisco Soria Melguizo, S.A., Spain) with shaking at 100 rpm at 37°C under microaerophilic conditions (5% CO_2_). E. coli strains were cultured on brain heart infusion (BHI) agar and BHI broth with shaking at 100 rpm at 37°C. The ampicillin MICs of the E. coli strains carrying the plasmid variants were determined using the broth microdilution method according to the CLSI guidelines (60). The usual 2-fold dilution was used until a concentration of 64 mg/L was achieved, and then we tested growth in the presence of ampicillin in multiples of 64 mg/L, to achieve smaller ranges between MIC values. In addition, for very high MIC levels, concentrations in 500-mg/L steps were used between 3,000 mg/L and 5,000 mg/L. The values displayed are the mean values of independent measurements. Ampicillin was provided by Sigma (Sigma-Aldrich Química S.A., Spain).

### Electroporation conditions.

Electrocompetent E. coli DH5α and H. influenzae RdKW20 strains were prepared as previously described (61, 62). The electrocompetent cells were transformed by electroporation with pB1000 from P. multocida BB1034 and with the rest of the pB1000 variants using a Gene Pulser apparatus (Bio-Rad, USA). Electroporation was performed using the following conditions: 2.5 kV/cm, 25 μF, and 200 Ω.

### Experimental evolution assays.

Nine experimental evolutions of E. coli DH5α with pB1000 were performed in two independent experiments. Transformation of DH5α was carried out via electroporation with pB1000 from P. multocida BB1034. For the first experiment, E. coli DH5α was transformed with pB1000, and one clone was selected, from which 3 replicate populations were derived (lineages 1 to 3; see Table 1). For the second experiment, we extracted fresh pB1000 plasmid DNA and transferred it to DH5α. Two clones were selected from this transformation, and each clone was used for 3 replicate populations (lineages 4 to 6 and lineages 7 to 9; see Table 1). For each of the resulting 9 populations, 10^6^ CFU of the bacteria bearing the plasmid were inoculated into 5 mL of BHI with a subinhibitory concentration of ampicillin of 50 mg/L. The cultures were incubated at 37°C and 100 rpm for 50 days (~500 generations), with a daily transfer of 5 μL of the culture into 5 mL of BHI with 50 mg/L ampicillin (representing 10 generations per day [63]). Before starting the evolution assays, plasmid DNA was analyzed by performing PCR and using Sanger sequencing. Every 5 days during the course of the assays, bacteria from lineages 4 to 9 were plated on BHI agar plates with ampicillin and frozen, and plasmid DNA was extracted using the QIAprep Spin miniprep kit (Qiagen, Inc., Germany). Additionally, in three lineages (7 to 9), pB1000 was extracted every 24 h during the first 5 days. The acquisition of ISs was validated by performing PCR amplification of the *shyP* gene. Further analysis of the extracted plasmid DNA is described in the “Sequencing” section of Material and Methods.

### Sequencing.

To analyze the plasmids during the evolution (six out of nine lineages) and at the end of the evolution (all nine lineages), we amplified the entire plasmid using PCRs with the primers pB1000.A F/R to pB1000.E F/R (Table 4). To amplify *oriV* and the region of the ISs, we used the primers pB1000 ori F/R (45) and pB1000.IS F/R, respectively. Reagents for the PCRs are described in the section “Plasmids and Plasmid Construction.” Sanger sequencing of the DNA fragments was carried out by Secugen S.L. (Spain) and Macrogen, Inc. (South Korea). To assess the relative frequency of variants with different SNPs in the same position, we studied the chromatograms obtained from sequencing with the software QSVanalyser (64).

**TABLE 4:** Primers used for constructions/sequencing

Primer name	Sequence
pBAD-3I F	GAATTCACCATGATAAGTACATCAAGCATAGC
pBAD-3I R	CCGGGTACTTATTATGTGAAAACATCAGAAATT
pBAD-3V F	TTCACATAATAAGTACCCGGGGATCCTCTAGAG
pBAD-3V R	ACTTATCATGGTGAATTCCTCCTGCTAGC
pBAD-40I F	GAATTCACCGTGATTTATGTGAAAACATCAG
pBAD-40I R	CCCGGGTACTTATTAGGCTAATTTATTTAAAACGATG
pBAD-40V F	TTAGCCTAATAAGTACCCGGGGATCCTCTAGAGTC
pBAD-40V R	ATAAATCACGGTGAATTCCTCCTGCTAGCC
pB1000.IS F	AGGGCATACAATGGGAAAGG
pB1000.IS R	GTGTGGCTGATTGTTGCACAG
pB1000 ori F	AATTGGTTGGACAATAACGCA
pB1000 ori R	AATCTCGCTTATCAGGTGTGC
pBAD43v_F	TAATAAGTACCCGGGGATCC
pBAD43v_R	ATAAATCACGGTGAATTCCTCCTGCTAGCC
pB1000.A F	TGTTCGAGCTGAACCGCATAG
pB1000.A R	GCTCTCTAATTCTTTCGATAA
pB1000.B F	CGACCCTAATCGCCTTGACGA
pB1000.C F	AGTGTTAGCAAGATGATCAGA
pB1000.C R	TTATCGTACACTTTCCA
pB1000.D R	AAATCAGCGGAGCCGATAGGC
pB1000.E F	TTAATACGAAAATTAAGCTC
pB1000.E R	GCCCTGCAGGATTTGGGCGGT
ORF40-3FT_F	GAATTCACCGTGATTTATGTGAAAACATCAG
ORF40_3FT_R	CCGGGTACTTATTACTTGTCATCGTCATCCTTGTAGTC

### Stability assays and fitness cost.

All tested plasmids were extracted after the evolution experiments and transformed again into naive E. coli DH5α. The stability was obtained by calculating the maintenance rate (*M*) of each plasmid variant. For this, 10^6^ CFU of each strain were inoculated into 5 mL of BHI and incubated for 24 h, and subsequently, 5 μL of the cultures were transferred daily into 5 mL of fresh BHI for 10 days (~100 generations). Every day, bacteria were plated on nonselective BHI-agar plates, and the proportion of resistant colonies was determined by replica plating of 100 colonies onto BHI agar plates with 50 mg/L of ampicillin. The ratio (*R*) between resistant and sensitive bacteria at each time point, divided by the same ratio at time zero, gave us the maintenance of the plasmid at each moment. The plasmid maintenance rate was then calculated as the slope of the linear regression model *M* = ln(*R*)/*t*, where time (*t*) was measured in bacterial generations, calculated as the log_2_ of the dilution factor.

The fitness cost of the plasmids was obtained by calculating the plasmid loss rate per 10 generations, in 3 to 9 independent experiments per strain, adapting the method described by San Millan et al. (27). In our case, we performed the assays within 2 days (instead of 6 days) to minimize the effect of the new mutations and segregational loss of plasmids. Note that the maintenance of pB1000 in E. coli is significantly lower than that of pB1000 with IS or mock fragment and is therefore reflected together with the fitness cost in the selection coefficient.

### Determination of plasmid copy numbers.

The copy number of each plasmid in E. coli DH5α was analyzed using quantitative PCR (qPCR), as described in reference 65, using a My iQ single-color real-time PCR detection system (Bio-Rad, USA). Each strain was grown in 5 mL of BHI to an optical density of approximately 0.9 at 600 nm (OD_600_), and DNA was extracted in triplicate using a QIAamp DNA minikit (Qiagen, Inc., Germany). DNA was quantified using a BioPhotometer (Eppendorf, Germany) and was digested with PstI (TaKaRa, Japan) to prevent the copy number from being underestimated (66). We designed a specific qPCR for the plasmid-carried *bla*_ROB-1_ gene (primers CCAATTCTGTTCATTCGGTAAC [forward] and CATAAGCAAAGCGTTCATCTG [reverse]; amplicon size, 195 bp) and for the monocopy chromosomal gene *uidA* (primers GTCAATAATCAGGAAGTG [forward] and AAAGAAATCATGGAAGTAA [reverse]; amplicon size, 201 bp) to compare the ratio of plasmid and chromosomal DNA. All reactions had an efficiency between 94 and 100%, with an r^2^ higher than 0.998. The efficiency of the reactions was calculated from the standard curve obtained by performing qPCR of serial dilutions of template DNA. qPCRs were performed using iQ SYBR green supermix (Bio-Rad) at a final DNA concentration of 10 pg/μL. The amplification conditions were as follows: initial denaturation for 10 min at 95°C, followed by 30 cycles of denaturation for 1 min at 95°C, annealing for 1 min at 58.5°C (*bla*_ROB-1_) or 55°C (*uidA*), and extension for 1 min at 72°C. To calculate the copy number of plasmids per chromosome we used the formula *cn* = [(1 + *Ec*)*^CTc^*/(1 + *Ep*)*^CTp^*] × (*Sc*/*Sp*), where *cn* is the plasmid copy number per chromosome, *Sc* and *Sp* are the sizes of the chromosomal and plasmid amplicons (in base pairs), respectively, *Ec* and *Ep* are the efficiencies of the chromosomal and plasmid qPCRs (relative to 1), respectively, and *CTc* and *CTp* are the threshold cycles of the chromosomal and plasmid reactions, respectively. To correct the plasmid copy number in the case of high plasmid loss, the proportion of plasmid-bearing cells of the cultures used for DNA extraction was determined. To do so, cultures were plated onto nonselective BHI agar plates, and the fraction of resistant colonies was assessed after replica plating of 100 colonies onto BHI agar plates with 50 mg/L of ampicillin. The corrected PCN is calculated as the ratio of the measured plasmid copy number and the percentage (relative to 1) of the resistant colonies (PCN/[resistant colonies/total number of colonies]).

### Plasmids and plasmid construction.

The plasmids that were used in this study are listed in Table 3, and the primers for construction are listed in Table 4. To clone ORF40 and ORF3 downstream of the promoter *araBAD* in pBAD43, the ORFs were amplified via PCR with the primers pBAD-3I F/R for the ORF3 insert and pBAD-3V F/R for the linearization of the pBAD43 vector. For the plasmid used in Western blotting, ORF40-3FT was cloned into pBAD43 using the primers pBAD43v_F/R and ORF40-3FT_F/R. All PCRs were carried out using reagents from Biotools (Biotools B&M Labs, S.A., Spain) and Phusion polymerase with its corresponding 5× Phusion HF buffer (Thermo Fisher Scientific, USA). PCR products were purified with the QIAquick PCR purification kit (Qiagen, Inc., Germany) and in case of extraction from gel with the QIAquick gel extraction kit (Qiagen, Inc.). For the pBAD43::ORF40 construction, pBAD-40I F/R were used for ORF40 amplification and primers pBAD-40V F/R for the linearization of the pBAD43 vector. Insert and vector fragments were assembled using the Gibson Assembly cloning kit (New England Biolabs, USA).

### Western blots.

To detect the peptide encoded by ORF40, we used the 3×Flag system (Sigma-Aldrich, USA) by cloning the 3×Flag tag sequence in the C-terminal position of the ORF40 into pBAD43 (pBAD43::ORF40-3FT). For protein extraction, we diluted an overnight culture of E. coli cultures harboring the pBAD43::ORF40-3FT plasmid 1:100 in fresh LB medium. The cultures were incubated at 200 rpm and 37°C either by repressing ORF40 expression using glucose (1%) or inducing it with arabinose (0.2%). At an OD_600_ of 0.6, we centrifuged 1 mL of the cultures for 5 min at 13,400 rpm and 4°C and resuspended the pellet in 60 μL of 1× Laemmli sample buffer. The samples were frozen immediately at −20°C. As a control for the detection of the Flag tag, we extracted proteins from E. coli populations carrying the empty vector (negative control) and the control Flag tag vector (positive control).

After thawing the samples, they were heated to 95°C for 10 min to denaturalize the proteins. A total of 10 μL of the samples, as well as 5 μL of Precision Plus Protein Western blotting standard (Bio-Rad, USA) were loaded to a Bolt 4 to 12% Bis-Tris Plus gel (Invitrogen, USA). Gel electrophoresis was set at 200 V for 30 min. Afterward, the proteins were transferred to a nitrocellulose membrane using the iBlot2 system (Invitrogen). The membranes were blocked using TBS-T (Tris-buffered saline [TBS] with 1% Tween 20 [PanReac, USA]) and 10% powdered milk (Nestlé Sveltesse, Switzerland) for 2 h and 100 rpm at room temperature. Then, the membranes were washed 3 times with TBS-T and incubated for 2.5 h and 100 rpm at room temperature with 1:500 of the primary monoclonal Anti-Flag M2 mouse antibodies (Sigma-Aldrich, USA) and 1:2,000 of monoclonal anti-DnaK [8E2/2] antibodies (Abcam, UK). After another washing step with TBS-T, the membranes were incubated for 1.5 h and 100 rpm at room temperature with 1:5,000 of the secondary goat anti-mouse IgG antibodies with horseradish peroxidase (HRP) and 1:10,000 of the Precision Protein StrepTactin-HRP conjugate (Bio-Rad).

Following another washing step with TBS-T, membranes were incubated for 2 min using the Pierce ECL Western blotting substrate (Thermo Fisher Scientific, USA).

For visualization, the program Image Lab (Bio-Rad, USA) was used, and the emitted luminescence was detected with the system ChemiDoc XRS+ (Bio-Rad).
