# Supplementary figures and images for: Insertion Sequences Determine Plasmid Adaptation to New Bacterial Hosts
Source: mBio. 2023 Apr 25;14(3):e03158-22. doi: 10.1128/mbio.03158-22 (PMC10294622; doi:10.1128/mbio.03158-22)

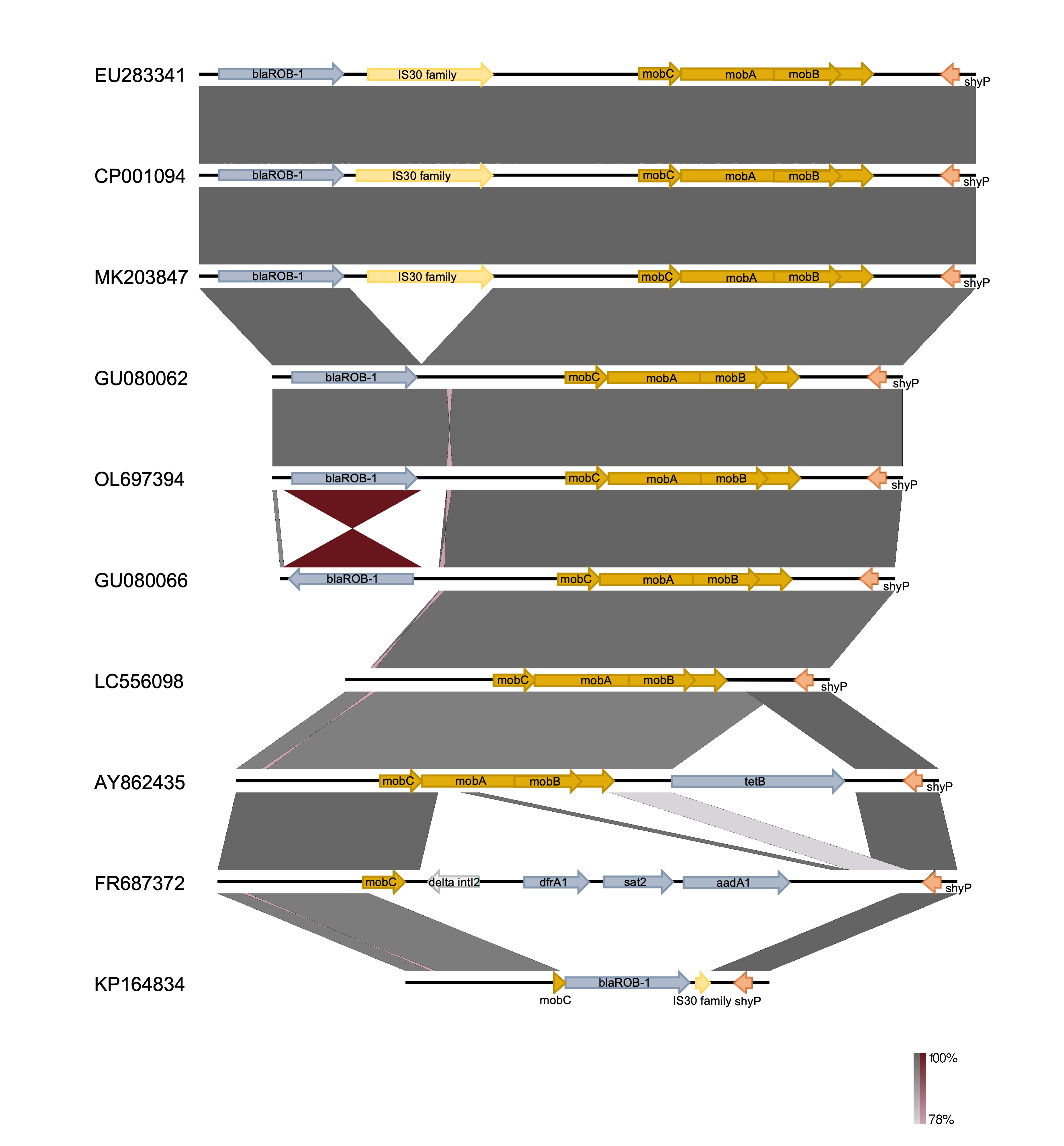

Supplement: FIG S1 [file mbio.03158-22-s0001.tif]

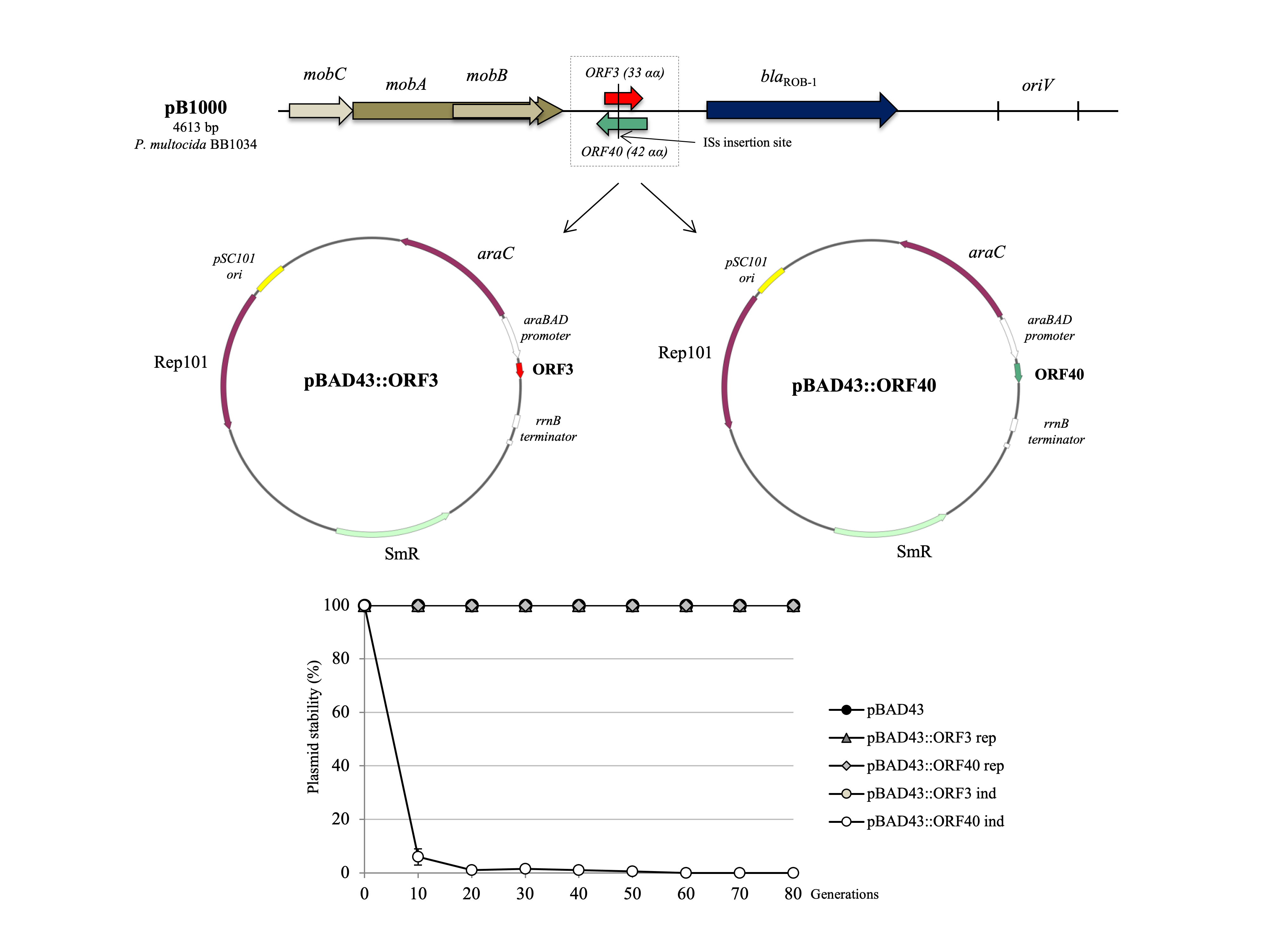

Supplement: FIG S2 [file mbio.03158-22-s0002.tif]

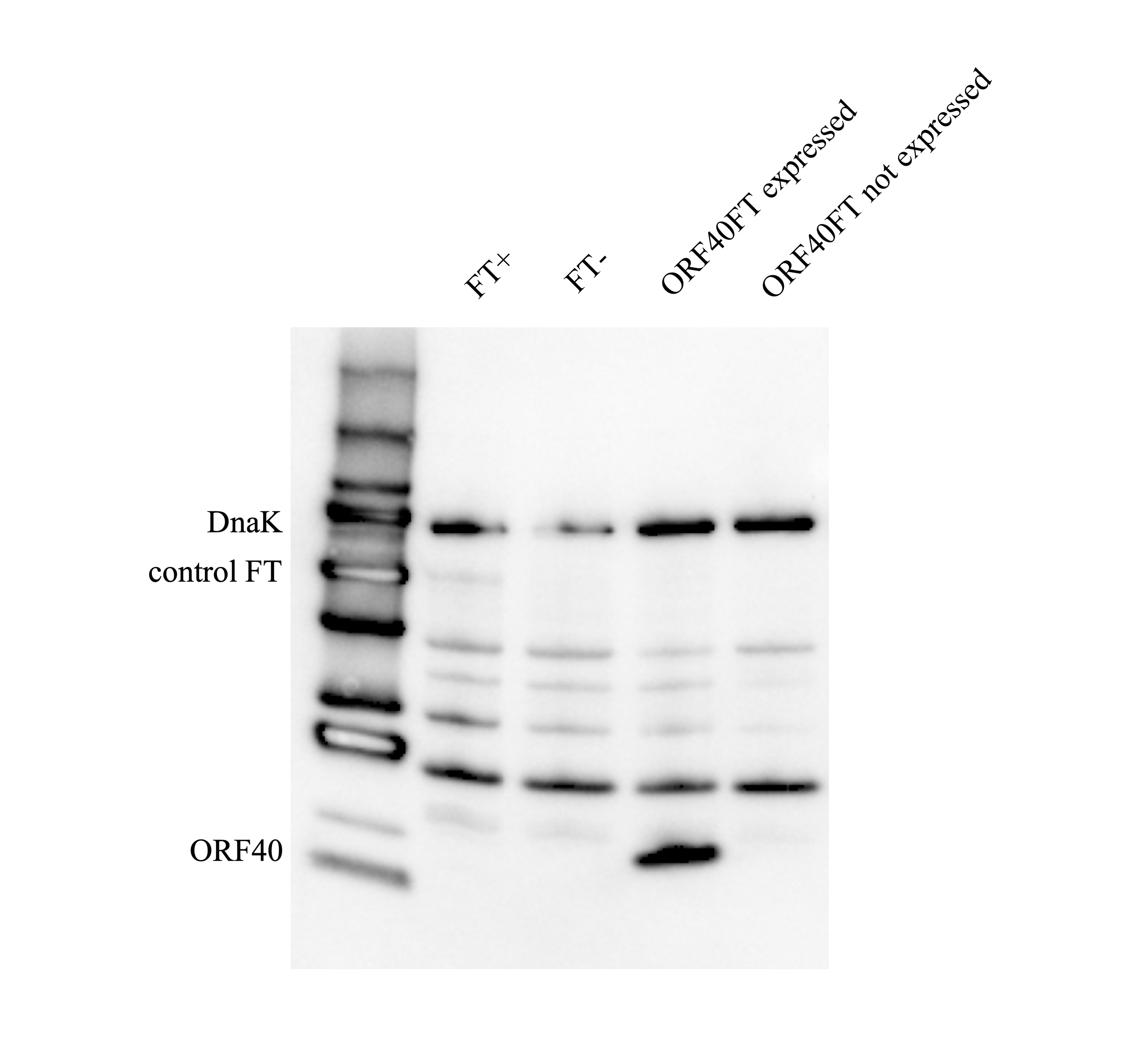

Supplement: FIG S3 [file mbio.03158-22-s0003.tif]
